# N-Cadherin cleavage during activated hepatic stellate cell apoptosis is inhibited by tissue inhibitor of metalloproteinase-1

**DOI:** 10.1186/1476-5926-2-S1-S8

**Published:** 2004-01-14

**Authors:** Frank Murphy, Julian Waung, Jane Collins, Michael JP Arthur, Hideaki Nagase, Derek Mann, R Christopher Benyon, John P Iredale

**Affiliations:** 1 Liver group, Division of Infection, Inflammation and Repair, Southampton University, Southampton General Hospital, Tremona Road, Southampton SO16 6YD, UK; 2 Mucosal Immunology, Southampton University, Tremona Road, Southampton, UK; 3 The Kennedy Institute, Imperial College, 1 Aspenlea Road, London W6, UK

## Abstract

Apoptosis of hepatic stellate cells (HSC) has previously been shown to occur during spontaneous resolution of experimental liver fibrosis. TIMP-1 has also been shown to have a key role because of its ability to inhibit apoptosis of HSC via matrix metalloproteinase (MMP) inhibition. This has led to further study of novel substrates for MMPs that might impact on HSC survival. N-Cadherin is known to mediate cell-cell contacts in fibroblasts. In this study we demonstrate that N-Cadherin is expressed by activated rat HSC. Furthermore, during apoptosis of HSC, the N-Cadherin is cleaved into smaller fragments. Apoptosis of HSC may be inhibited by TIMP-1. This is associated with reduced fragmentation of N-Cadherin. N-Cadherin may have an important role in supporting HSC survival while N-Cadherin cleavage may play a part in promoting HSC apoptosis in recovery from liver fibrosis.

## Introduction

The activated hepatic stellate cell plays a key role in liver fibrosis [1]. During spontaneous recovery from experimental liver fibrosis loss of activated hepatic stellate cells (HSCs) has been demonstrated to be through apoptosis or programmed cell death [2,3]. This has highlighted control of HSC apoptosis for further investigation. During spontaneous resolution of experimental liver fibrosis there is a change in the balance between matrix metalloproteinase (MMP) activity and their endogenous inhibitor TIMP-1. This change favours matrix degradation. During resolution of fibrosis there is a great reduction in TIMP-1 expression. Correlating with this event there is a fall in the numbers of activated HSCs. This observation was recently studied in depth in vitro. TIMP-1 reduced apoptosis of culture activated HSC in a dose dependent manner. Inhibition of HSC apoptosis by TIMP-1 has previously been shown to be due to its action of MMP inhibition [4]. This has led to the further study of potential MMPs and MMP substrates on the cell surface that might impact on HSC survival.

N-Cadherin, which mediates cell to cell contact, is expressed in activated HSC. It has been shown to signal via the beta-catenin pathway. Disruption of this interaction with blocking antibodies has been shown to promote apoptosis in melanoma cells [5]. Furthermore, MMP inhibitors including TIMPs have been demonstrated to promote fibroblast adhesion through stabilisation of focal adhesion contacts and up regulation of Cadherin function in vitro [6]. We studied the fate of N-Cadherin in HSC during apoptosis induced by cycloheximide or gliotoxin. We have demonstrated that activated rat HSC express intact 135 kDa N-Cadherin and that during apoptosis of these cells the N-Cadherin is degraded into smaller fragments. We have also demonstrated that TIMP-1 is able to reduce apoptosis of activated HSC promoted into apoptosis by cycloheximide. TIMP-1 treated rat HSC demonstrated reduced fragmentation of N-Cadherin.

## Methods

### Isolation of rat HSC

Rat HSC were extracted by pronase and collagenase digestion and purified by centrifugal elutriation as previously described [7]. Extracted rat HSC were cultured on plastic for 7–10 days in the presence of serum until activated.

### Stimulation of HSC apoptosis and examination of nuclear morphology by acridine orange

Apoptosis of activated rat HSC was induced by exposure to gliotoxin (1.5 micromolar) or cycloheximide (50 micromolar) in the presence of TIMP-1 (5 nanomolar) or a mutated TIMP-1 (T2G N-TIMP-1 with no MMP inhibitory activity: kind gift of Professor Hideaki Nagase). Following a 4 hour incubation at 37–C, nuclear morphology was assessed by adding acridine orange to each well (final concentration 1 –g/ml) and examining the cells under blue fluorescence. The total number of apoptotic cells were counted and expressed as a percentage of the total number of cells.

### Quantification of HSC apoptosis by Caspase-3 activity assay

To validate the acridine orange assay, HSC apoptosis was further assessed by a colorimetric assay for Caspase-3 activity (Calbiochem) following the manufacturer's instructions.

### Western blotting for N-Cadherin-Rat

HSC protein extracts from different conditions were studied by Western blotting for N-Cadherin using a mouse monoclonal antibody to an intracellular epitope of N-Cadherin (Clone 3B9, Zymed, California). Normalised protein extracts (10 micrograms) were separated on 12% SDS PAGE gels. The samples were electrotransferred onto polyvinylidene difluoride membrane. This was blocked using 1% non fat dry milk in TBS for 1 hour. The membranes were incubated overnight with the primary antibody (1:100 dilution) or a non immune control IgG as a negative control. The membranes were washed three times in 0.1% Tween TBS (TTBS) before addition of the secondary antibody (rabbit anti mouse horseradish peroxidase at 1:2000 dilution) in 0.5% non fat dry milk TBS for 1 hour. After a further two washes with TTBS then water, the membranes were developed using ECL (Amersham Biosciences) and autoradiography according to the manufacturer's instructions.

## Results

### Wild type TIMP-1 but not the inactive T2G mutant TIMP-1 reduces apoptosis induced by cycloheximide

Both acridine orange staining and Caspase-3 activity assay demonstrated that the wild type TIMP-1 but not the T2G mutant TIMP-1 significantly inhibited HSC apoptosis induced by exposure to cycloheximide for 4 hours. This suggested that the inhibitory effect of TIMP-1 on rat HSC apoptosis was via effects on MMP inhibition rather than an alternative mechanism.

### Cyclohexmide and gliotoxin both promote apoptosis of rat HSC

Exposure of rat HSC to cycloheximide or gliotoxin promoted apoptosis of these cells when assessed by acridine orange staining and was further confirmed by increased caspase-3 activity over untreated cells.

### Rat activated HSC express intact 135 kDa N-Cadherin

Western blotting of rat activated HSC protein extracts clearly demonstrated an intact 135 kDa form of N-Cadherin. Furthermore, 60 and 38 kD truncated forms of N-Cadherin were observed in protein extracts from rat activated HSC cultured in serum.

### Cleavage of N-Cadherin observed during HSC apoptosis

Cleavage of N-Cadherin was demonstrated with the appearance of a 60 kDa fragment after 3–4 hours exposure to cycloheximide or gliotoxin. This fragmentation was reduced by co-incubation with 5 nM recombinant TIMP-1 but not by the non-functional T2G mutant TIMP-1.

### Bioinformatic analysis of rat N-Cadherin reveals multiple potential MMP cleavage sites

Analysis of the known amino acid sequence of rat N-Cadherin reveals 19 potential cleavage sites for MMPs. This suggests that MMPs have a role at least in part in pericellular proteolysis that occurs during apoptosis of culture activated rat HSC.

## Discussion

Previous studies have shown that TIMP-1 is able to reduce apoptosis of cultured rat and human HSC. This effect is thought to be because of its primary function as an inhibitor of matrix metalloproteinases [4]. This observation has led to the further study of which MMP and which substrates might be important in mediating HSC survival or apoptosis. This work has examined the fate of the cell surface protein N-Cadherin that is known to be up regulated during HSC activation. The activated rat HSC express intact 135 kDa N-Cadherin that is degraded into smaller fragments during apoptosis induced by gliotoxin or cycloheximide. N-Cadherin fragmentation was reduced by exposure to TIMP-1 but not a non functional mutant TIMP-1 (the T2G mutant TIMP-1). Protein database analysis indicated that rat and human N-Cadherin contain a number of potential MMP cleavage sites. MMP inhibitors have previously been demonstrated to promote survival of fibroblasts through up regulation of N-Cadherin function [6]. MMP cleavage of N-Cadherin may be an important mechanism promoting HSC apoptosis in recovery from liver fibrosis.
